# Analytical technologies to revolutionize the environmental mutagenesis-and genome- research -from the basics to the cutting-edge research-: the Open Symposium of the Japanese Environmental Mutagen and Genome Society (JEMS), 2022

**DOI:** 10.1186/s41021-023-00265-6

**Published:** 2023-03-09

**Authors:** Naoki Koyama, Akira Sassa

**Affiliations:** 1 Global Drug Safety, Eisai Co., Ltd., 5-1-3 Tokodai, Tsukuba-shi, Ibaraki, 300-2635 Japan; 2 Department of Biology, Graduate School of Science, Chiba University, 1-33 Yayoi-cho, Inage-ku, Chiba, 263-8522 Japan

**Keywords:** Machine learning model, In silico mutagenicity, xenoBiotic, DNA damage, Directed evolution, Single-cell DNA replication sequencing, Mutagenesis, Carcinogenesis, Environmental mutagen

## Abstract

The Open Symposium of the Japanese Environmental Mutagen and Genome Society (JEMS) entitled “Analytical technologies to revolutionize the environmental mutagenesis and genome research -From the basics to the cutting-edge research-” was held online, on June 11th, 2022. The purpose of this symposium was to provide an opportunity to highlight the cutting-edge research for measurement technologies, and informational and computational (in silico) sciences for the purpose of applying them to deepen scientific knowledge and better understanding the relationship between genes and environmental mutagens. These advanced technologies and sciences are indispensable for the prediction of pharmacokineticses, mutagenicities of chemical substances, and structures of biomolecules including chromosomes. In this symposium, we invited six scientists who are continuing to expand the frontiers in the fields of health data science. Herein, the organizers present a summary of the symposium.

## Background

The Open Symposium of the Japanese Environmental Mutagen and Genome Society (JEMS) is organized annually to present the cutting-edge technologies and sciences related to the fields of genetic toxicology and environmental mutagenesis to the public, and the proceedings are summarized in the meeting reports [1–6]. Last year (2021), the symposium was held, entitled “Genome editing technologies and our environment”, on genome editing technology including the “CRISPR-Cas9 system and was organized by Drs. Isao Kuraoka, Katsuyoshi Horibata, Akira Sassa, and Shuichi Masuda. In 2022, the symposium, “Analytical technologies to revolutionize the environmental mutagenesis and genome research - From the basics to the cutting-edge research” was designed by 6 scientists, among them were JEMS members and non-members, to present their work at the symposium. The aim of the symposium this year was to provide an opportunity to highlight the cutting-edge research for measurement technologies, informational and computational (in silico) sciences, their application to deepen scientific knowledge, and to understand the relationship between genes and environmental mutagens. These advantaged technologies and sciences are indispensable for our research, such as the prediction of pharmacokineticses and mutagenicities of chemical substances, structures of biomolecules including chromosomes. The symposium was held online on June 11th, 2022. Through this report, the organizers present a summary of the event and presentations.

## Symposium program

Masami Yamada (President, JEMS: National Defense Academy of Japan): Inaugural speech.

Akira Sassa (Chiba University): Introduction

Session 1 (Chair: Naoki Koyama)

Tsuyoshi Esaki (Shiga University): Prediction of drug properties to accelerate data-driven drug discovery -From data collection to prediction-.

Toshihiko Sawada (xenoBiotic Inc.): Development of Ames mutagenicity predictor xenoBiotic using quantum chemical calculations.

Naoki Koyama (Eisai Co., Ltd.): (Q)SAR/AI-based genotoxicity assessment in healthcare company.

Session 2 (Chair: Akira Sassa)

Ai Suzuki (Tohoku University): Structural analysis of hydration water around damaged DNA by using quantum chemical molecular dynamics.

Yutaka Saito (National Institute of Advanced Industrial Science and Technology): Machine-learning-guided directed evolution for functional protein design.

Ichiro Hiratani (RIKEN): The 3D genome and the 4D nucleome research – where they are, where they might go, and the future of single-cell genomics.

Naoki Koyama (Eisai): Concluding speech.

## Meeting report

Dr. Tsuyoshi Esaki presented advanced research results in pharmacokinetic properties and machine learning-based toxicity prediction (e.g., hERG). In addition, he gave a lecture on the characteristics prediction and the essence of utilizing data analysis techniques in predicting user performance. Drug discovery is costly and time-consuming. He explained that it is important to develop a machine learning model for resolving these issues that utilizes several data such as structured data and properties, to predict the properties of candidate drugs, aiming to improve efficiency and productivity in research and development. If we can predict the physicochemical properties of drugs from the structural data in library compounds, it will be possible to reduce the scale of the studies and to identify candidate compounds, which can be expected to reduce the cost and time required for research and development.

Dr. Toshihiko Sawada gave a talk on a case study of predicting mutagenicity using quantum chemical calculations. In addition, the Ames mutagenicity prediction software ‘xenoBiotic’, which is under development, was introduced, and prospects were discussed. The Mammalian Mutagenicity Study Group (MMS) of the JEMS proposed the collaborative study group for a local QSAR model to support expert judgments on aromatic amine mutagenicity based on quantum chemical calculations. It is expected that the results of the collaborative study including ‘xenoBiotic’ will contribute to the development of mutagenicity prediction.

Dr. Naoki Koyama presented the Ames mutagenicity prediction system ‘YosAI’ jointly developed with SBX Corporation. ‘YosAI’ increases the prediction accuracy and efficiency of mutagenicity evaluation by integrating our expert knowledge. In addition, AI technology based on internal/external Ames data, and commercial prediction software, CASE Ultra. This system can be used (1) to search for the mutagenicity and carcinogenicity data, (2) to display the CASE Ultra structure alerts, (3) to search for similar structures, and (4) to show the presence or absence of electrophilicity for DNA binding ability. Thus, the YosAI system follows the expert review flow recommended in the ICH M7 guidelines. Furthermore, YosAI also implements the Artificial Neural Network method that uses parameters, such as structure alert and DNA binding ability with 93% accuracy [7]. This hybrid knowledge/statistical- model is expected for use in healthcare companies.

Dr. Ai Suzuki focused on the mechanism of DNA damage recognition by human 8-oxoguanine DNA glycosylase (hOGG1) and presented the in-silico analysis to assess the structure and function of water molecules around 7,8-dihydro-8-oxoguanine (8-oxoG). Due to the presence of an oxygen atom at the C8 position of 8-oxoG, the first layer of water molecules around the 8-oxoG may exert electrostatic and structural effects, which mediates the initial recognition step of the damaged DNA by hOGG1. Using the accelerated quantum chemical molecular dynamics method, Dr. Suzuki revealed the altered electronic configuration around a single or tandem 8-oxoG embedded into DNA with surrounding water molecules. The study can contribute to a better understanding of the mechanism of the initial lesion-recognition step during oxidative DNA damage repair.

Dr. Yutaka Saito gave a talk on a “directed evolution” for protein engineering of enzymes producing useful materials, biopharmaceuticals, and fluorescent proteins essential to life science studies. He presented the development of the machine-learning-guided mutagenesis platform that combines molecular evolution with machine learning. Based on this methodology, his group reported the effectiveness of directed evolution by machine learning with their latest insight into its regulatory mechanism. The design of biomolecules including, proteins, DNA, and mRNA using the novel approach enables the acceleration of discovery of new functional proteins.

Dr. Ichiro Hiratani presented his research on the single-cell DNA replication sequencing (scRepli-seq) method, which allows for the genome-wide observation of replication timing in single cells by next-generation sequencing. Replication timing of the genomic DNA is correlated with the chromatin dynamics and chromosome architecture, thus defects in replication timing are thought to reflect abnormalities in higher-order genome organization. This technique enables the elucidation of subnuclear compartment profiles during cell differentiation at the single-cell level. Further, scRepli-seq can be utilized to detect chromosomal aberrations in mouse embryos, providing valuable insight into genome integrity.

The symposium was held virtually on the Zoom webinar platform, which allowed our participants to communicate with each other through chats. Approximately 120 participants joined this symposium, and a questionnaire survey revealed that 30% of the attendees were not JEMS members. As the organizers, we would like to thank everyone who joined this symposium.

## Data Availability

Not applicable.

## Abbreviations

JEMS: Japanese Environmental Mutagen Society
MMS: Mammalian Mutagenicity Study group
hOGG1: human 8-oxoguanine DNA glycosylase
8-OxoG: 7,8-dihydro-8-oxoguanine
scRepli-seq: single-cell DNA replication sequencing
QSAR: Quantitative Structure Activity Relationship
